# Antiquity of Mercury

**Published:** 1887-08

**Authors:** 


### Antiquity of Mercury.

Quicksilver, known to the Plinies and Dioscorides, was first authentically used by Avicenna and by Paracelsus. The first mention, however, of calomel we find in the seventeenth century, when it was known under various synonyms, such as aquila alba (the white eagle), leo mitigatus, drago mitigatos, panchymagogum, or mineral manna. It has held its own ever since, and is one of the first on the list of the present armamentorium.

